# Neutrophil dysfunction predicts 90‐day survival in patients with acute on chronic liver failure: A longitudinal case–control study

**DOI:** 10.1002/jgh3.12344

**Published:** 2020-05-05

**Authors:** Kunaal Makkar, Shallu Tomer, Nipun Verma, Sahaj Rathi, Sunil K Arora, Sunil Taneja, Ajay Duseja, Yogesh K Chawla, Radha K Dhiman

**Affiliations:** ^1^ Department of Internal Medicine Post Graduate Institute of Medical Education and Research Chandigarh India; ^2^ Department of Immunopathology Post Graduate Institute of Medical Education and Research Chandigarh India; ^3^ Department of Hepatology Post Graduate Institute of Medical Education and Research Chandigarh India

**Keywords:** acute decompensation, acute on chronic liver failure, cirrhosis, immune dysfunction, neutrophil dysfunction

## Abstract

**Background and Aim:**

Innate immune disarray is a key component in the development and progression of acute on chronic liver failure (ACLF) and predisposition to infections. We evaluated the neutrophil dysfunction and its impact on outcomes in patients with ACLF.

**Methods:**

Forty patients with acute decompensation of cirrhosis (10 each of grades 0, 1, 2, and 3 ACLF) and 10 healthy controls were prospectively evaluated for neutrophil immunophenotype (NP), neutrophil phagocytic capacity (NPC), and oxidative burst (OB) in both resting and stimulated conditions. The patients were followed up for 90 days or until death or transplant, whichever was earlier.

**Results:**

NP was normal (in %) and NPC (in mean fluorescence intensity [MFI]) was better in controls compared to patients with ACLF (83.74 ± 12.38 *vs* 63.84 ± 22.98; *P* = 0.007 and 98.33 ± 130.60 *vs* 18.73 ± 17.88, *P* = 0.001, respectively). Resting OB was higher in patients with ACLF compared to controls (97 ± 4.9% *vs* 91 ± 9%; *P* = 0.034), but it failed to increase further after stimulation, suggesting an immune exhaustion. NP was normal (in %) and NPC (in MFI) was better in 90‐day survivors compared to nonsurvivors (78 ± 11.9 *vs* 62.2 ± 24.11, *P* = 0.02 and 33.3 ± 22.7 *vs* 16.36 ± 13.3; *P* = 0.004, respectively). Phenotypically normal neutrophils >71.7% had 78.6% sensitivity and 65.4% specificity with an area under receiver operating curve (AUROC) of 0.70 (95% confidence interval [CI]: 0.55–0.90); *P* = 0.017, and NPC >17.32. MFI had 71.4% sensitivity and 69.6% specificity with an AUROC of 0.73 (95% CI: 0.54–0.86), *P* = 0.035, in predicting 90‐day survival.

**Conclusion:**

Neutrophils have impaired bactericidal function in patients with ACLF compared to healthy adults. Neutrophil phenotype and phagocytic capacity may be used to predict 90‐day survival in patients with ACLF.

## Graphical abstract

Neutrophils have impaired bactericidal function in patients with ACLF as compared to healthy adults. Neutrophil phenotype and phagocytic capacity may be used to predict 90‐day survival in patients with ACLF.

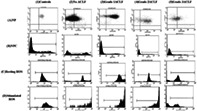

## Introduction

Acute on chronic liver failure (ACLF) is a clinical syndrome of acute deterioration of liver functions in patients with pre‐existing chronic liver disease. It is characterized by the presence of hepatic and/or extrahepatic organ failures, leading to high mortality rates.^1^ Despite being recognized for almost two decades, the management of such patients continues to be a major challenge.^2^

Patients with ACLF often have infection initiating or complicating the disease process, which increases the associated morbidity and mortality.^3^ Whether infections are a precipitating factor for ACLF or a consequence of immune dysfunction continues to be a matter of debate.^2^ Patients with ACLF are postulated to be in a state of immune disarray between a systemic hyperinflammatory state responsible for intense immune activation and an anti‐inflammatory milieu or immune exhaustion where the patient is prone to developing infections.^4^ Identifying where the patient lies on this spectrum can be crucial in planning the treatment of these patients.

Neutrophils are an important part of the innate immune system.^5^ They are responsible for killing pathogens by priming and chemotaxis followed by phagocytosis, degranulation, extracellular traps, and generating reactive oxygen species.^6^ Each step of their functions has been found to be dysfunctional in patients with ACLF. Paradoxically, activated neutrophils have also been shown to induce liver injury in various experimental models ranging from sepsis^7^ to alcoholic hepatitis.^8^ Neutrophils may have an exaggerated burst activity coupled with an impaired phagocytosis,^9^ thereby leading to increased inflammation but poor killing of the pathogens in patients with ACLF. Thus, they may act as a double‐edged sword responsible for the infections and the development of liver injury in patients with ACLF.^10^ The data on neutrophil dysfunction and its impact on clinical outcomes in patients with ACLF is scarcely reported. Thus, we aimed to characterize neutrophil dysfunction in order to better understand the pathogenesis of immune dysfunction and its role as a prognostic marker in patients with ACLF.

## Methods

#### *Patient selection*

Forty patients with acute decompensation (AD) of cirrhosis were enrolled consecutively on admission at the Department of Hepatology, Postgraduate Institute of Medical Education and Research (PGIMER), Chandigarh, India, from May 2016 to April 2017. AD of cirrhosis was defined as per the CANONIC study.^11^ ACLF was further subclassified as per chronic liver failure–sequential organ failure assessment (CLIF‐SOFA) scores depending on the number of organ failures: no ACLF (ACLF‐0, no organ failure, or single‐organ failure in patients with serum creatinine levels <1.5 mg/dL and no hepatic encephalopathy or single cerebral failure in patients with serum creatinine levels <1.5 mg/dL), grade 1 (ACLF‐1, single kidney failure or single‐organ failure in patients with serum creatinine levels ranging from 1.5 to 1.9 mg/dL, and/or grades 1–2 hepatic encephalopathy or single cerebral failure in patients with serum creatinine levels ranging from 1.5 to 1.9 mg/dL), grade 2 (ACLF‐2, Two organ failures), and grade 3 (ACLF‐3, three or more organ failures).^11^

#### *Study design*

A longitudinal prospective case–control study was conducted. Patients with AD of cirrhosis with ACLF‐0 (*n* = 10), ACLF‐1 (*n* = 10), ACLF‐2 (*n* = 10), and ACLF‐3 (*n* = 10) were prospectively enrolled. Neutrophil phenotype (NP), neutrophil phagocytic capacity (NPC), and oxidative burst (OB) (spontaneous and stimulated with phorbol 12‐myrisate13‐acetate‐PMA) were determined and compared to 10 controls. Baseline sampling was performed within 24 h of admission to hospital. Active infection was ruled out in patients by clinical and laboratory investigations as outlined in exclusion criteria.

#### *Inclusion criteria*

Patients with AD of cirrhosis between the ages of 18 and 70 years were included. Healthy age‐ and gender‐matched volunteers with no history of liver disease were used as controls. The alcohol intake in controls was <20 g/day in the last 3 months, and participants had not taken alcohol or exercised excessively in the 24 h prior to blood being drawn.

#### *Exclusion criteria*

Patients excluded were those with active infection (on either clinical, laboratory, or radiological investigations), active malignancy, uncontrolled diabetes mellitus, seropositivity for human immunodeficiency virus, patients on immunosuppressants such as steroids, other known immunodeficiencies, and prior decompensation or episode of ACLF in the last 3 months.

The following criteria were used for classifying infections.

1. Spontaneous bacteremia: positive blood cultures without a source of infection;
2. Spontaneous bacterial peritonitis: ascitic fluid polymorphonuclear cells >250/μL, with or without positive culture.
3. Lower respiratory tract infections: new pulmonary infiltrate in the presence of: (i) at least one respiratory symptom (cough, sputum production, dyspnea, pleuritic pain) with (ii) at least one finding on auscultation (rales or crepitation) or one sign of infection (core body temperature >38°C or less than 36°C, shivering, or leucocyte count >10 000/mm^3^ or <4000/mm^3^);
4. Bacterial enterocolitis: diarrhea or dysentery with a positive stool culture or *Clostridium difficile* toxin;
5. Clinical evidence of skin/soft tissue infection: fever with cellulitis;
6. Urinary tract infection: urine white blood cell >5/high power field with either positive urine Gram stain or culture;
7. Intra‐abdominal infections: diverticulitis, appendicitis, cholangitis etc.;
8. Any other infections, including fungal, as adjudged by the treating team and not covered above.

#### *Consent and data collection*

The study was performed in accordance with the Declaration of Helsinki, and ethical permission was obtained from the Ethics Committee of PGIMER Chandigarh. Informed consent was obtained from all participants. Clinical, biochemical, and physiological data were noted prospectively for 90 days or until death or transplant. Data included alcohol use, arterial ammonia (μmol/L), serum sodium levels (meq/L), arterial blood gas, and total and differential leukocyte count. Several organ failure scores were also quantified, including the Model of End‐stage Liver Disease score (MELD), CLIF‐SOFA, and chronic liver failure‐organ failure score (CLIF‐OF). Whole blood samples (3 mL) from the peripheral or central vein of patients or controls were collected aseptically in sterile Becton Dickinson heparinized vacutainers at enrollment and analyzed immediately or within 1 h under aseptic conditions for neutrophil function analysis.

#### *Immunophenotyping of neutrophils (NP)*

Neutrophils were analyzed for expression for CD16 and CD66B, and the proportion of cells that were positive for both CD antigens were considered phenotypically normal neutrophils. This was based on the study by Lakschevitz *et al*.,^12^ which showed that a combination of CD11b, CD16, and CD66b was able to determine pure neutrophil population irrespective of cell location, level of activation, and disease state. In another study,^9^ among ACLF patients, CD11b‐positive cells were similar between controls and ACLF. Thus, due to limited resources, we studied CD16 and CD 66b to identify a unique neutrophil signature.

Briefly, 100 μL of whole blood was taken in Falcon polystyrene test tubes. Red Blood Cell (RBC) lysis was conducted using a 1× RBC lysis buffer. Tubes were then centrifuged at 1500 rotations per minute (rpm) for 5 min, and the supernatant was discarded, leaving a neutrophil pellet at the bottom. Cells were washed with sterile phosphate‐buffered saline (PBS). Surface staining was performed by adding anti‐CD66B‐phycoerythrin and anti‐CD16–allophycocyanin and was incubated for 15 min in the dark at room temperature. The cells were then resuspended in sheath fluid and assessed on a flow cytometer (BD Calibur, San Jose, CA, USA). The percentage of cells that were positive for both CD66B and CD16 was gated, and analysis was performed using Cellquest software (BD Biosciences).

#### *Ex vivo neutrophil function studies*

All ex vivo studies were performed in pyrogen‐free conditions. Neutrophil function was examined in fresh neutrophils isolated from whole blood to more closely resemble physiological conditions. Neutrophil function studies were performed as described below.

#### *Neutrophil phagocytic capacity (NPC)*

Fluorescein isothiocyanate‐labeled opsonized IgG latex beads (Cayman's Phagocytosis Assay Kit) were used to measure phagocytosis. Heparinized whole blood was taken in two Falcon polystyrene test tubes. In the first tube, only blood was added, which served as control. In the second tube, blood was mixed with fluorescein‐labeled opsonized latex IgG beads and incubated at 37°C for 20 min. Fluorescence of IgG beads was quenched using ice‐cold trypan blue solution. Red blood cells were lysed using1× RBC lysis buffer. Samples were centrifuged at 1500 rpm for 5 min, and the supernatant was discarded, leaving a neutrophil pellet at the bottom of the tube. Then, neutrophils were washed with sterile PBS solution. Cells were stained with anti‐CD16 and anti‐CD66B antibodies and gated on forward‐ and side‐scatter graphs to measure phagocytic capacity. NPC was assessed by quantifying the total number of IgG beads phagocytized per neutrophil using mean fluorescence intensity (MFI).

#### *Oxidative burst (OB)*

**Figure 1:**
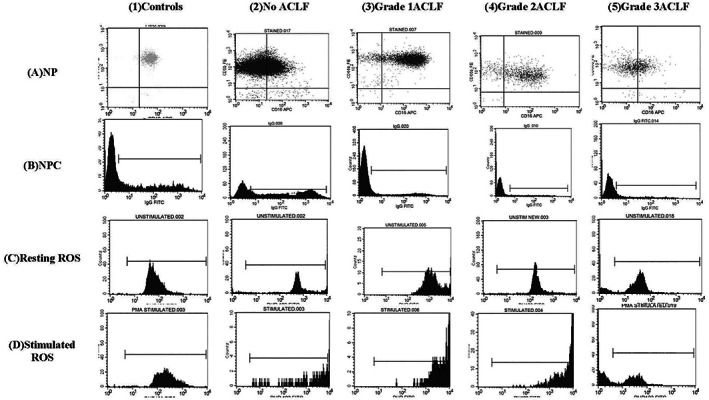
Side‐scatter graphical representation of neutrophil dysfunction on fluorescence‐activated cell sorting (FACS) in patients with various grades of acute on chronic liver failure (ACLF). (a) Neutrophil phenotype (NP): Percentage of cells positive for both CD16 and CD66B. (b) Neutrophil phagocytic capacity (NPC): Fluorescein isothiocyanate–labeled opsonized IgG beads phagocytized per neutrophil using mean fluorescence intensity (MFI). (c,d) Neutrophil oxidative‐burst: Percentage of cells producing reactive oxygen species with or without stimulation with phorbol 12‐myrisate13‐acetate (PMA).

Neutrophil reactive oxygen species (ROS) were quantified by burst test, which was used to determine the percentage of cells producing ROS with or without stimulation. Blood was placed in two Falcon polystyrene test tubes. The first tube had only blood, serving as control, while in other tube, PMA, which is an active principle of croton oil and a protein kinase C activator, was added to serve as stimulant.^13^ The production of ROS was measured using oxidation of dihydrorhodamine−123 to rhodamine, which produces green fluorescence. Red blood cells were lysed with lysing solution. Samples were centrifuged at 1500 rpm for 5 min, and the supernatant was discarded, leaving a neutrophil pellet at the bottom of the tube, which was washed with sterile PBS. OB was quantified as per the percentage of CD16 and CD66B neutrophils producing ROS using forward‐ and side‐scatter graph (Fig. 1).

### *Statistical analysis*

Continuous data were expressed as mean with SD and categorical data as count or frequency (percentage). Normal distribution as a continuous variable was analyzed using a *t*‐test; variables that were not normal were analyzed using the Mann–Whitney *U* test. Comparison between two groups was performed by independent t‐test. Discrete variables were analyzed using *χ* ^2^ test. Nonparametric AUROC analyses were performed for neutrophil phenotype and neutrophil phagocytic capacity. All statistical analyses were two‐sided and were performed at a significance level of 0.05. Statistical Package for the Social Sciences (SPSS), version 19 (IBM Corporation, Chicago, IL, USA) was used for statistical computations. Sample size was calculated by considering resting OB in ACLF patients to be 75% and in controls to be 10%,^14^ with an alpha error of 5% and power of 20% that required 10 patients in each group.

## Results

#### *Patient characteristics*

Forty patients of AD of cirrhosis were included in the study. As per CLIF‐SOFA criteria, 10 patients of each of the following groups were recruited: no‐ACLF and ACLF grades 1, 2, and 3. Patients had a mean age of 46.3 ± 10.0 years, and the majority (87%) was males. Patients had a mean MELD score of 27.5 ± 8.7 and a mean CLIF‐SOFA score of 11.3 ± 2.7. Most common causes of cirrhosis were alcohol (75%) and autoimmune hepatitis (10%). The most common precipitating event for ACLF was active alcoholism, seen in 50% patients, while in 25%, no precipitating event could be identified (Tables 1 and 2). Patients with ACLF had higher leucocyte count (14.9 ± 12.2 *vs* 9 ± 3.9 × 10^3^/mm^3^; *P* = 0.02) compared to the no‐ACLF group, and compared to controls, ACLF patients had a higher leucocyte (14.9 ± 12.2 *vs* 7.1 ± 1.7 10^3^/mm^3^ *P* = 0.03) and polymorph count (10.7 ± 10.6 *vs* 3.9 ± 1.4 × 10^3^/mm^3^; *P* = 0.001).

**Table 1:** Characteristics of study population

Age, years (Mean, SD)	46.3 ± 10.0
Gender (Male, %)	35 (88%)
Prior decompensation (%)	14 (35%)
Etiology of cirrhosis (%)	
Alcohol related	30 (75%)
HBV + alcohol	2 (5%)
HCV + alcohol	2 (5%)
Autoimmune	4 (10%)
NASH	2 (5%)
Others	1 (2.5%)
Acute precipitating event (%)	
Active alcoholism	20 (50%)
Upper gastrointestinal bleed	6 (15%)
Hepatitis B virus flare	2 (5%)
Unknown	10 (25%)
Hemoglobin, gm/dL (Median, IQR)	9.0 (8.0–10.7)
Platelet × 10^3^/mm^3^ (Median, IQR)	100 (53.5–173.5)
TLC × 10^3^/ mm^3^ (Median, IQR)	10 (7–15)
Polymorphs % (Median, IQR)	74.8 ± 10.3%
Creatinine, mg/dL (Median, IQR)	1.4 (1.1–1.9)
Bilirubin, mg/dL (Median, IQR)	12.3 (4.8–27.8)
Ammonia, μmol/L (Mean, SD)	129.8 ± 45.8
Organ failures (%)	
Liver	23 (76.6%)
Coagulation	14 (46.6%)
Kidney	13 (43.3%)
Cerebral	7 (23.3%)
Circulation	6 (20%)
Respiratory	4 (13.3%)
CLIF–SOFA score (Median, IQR)	11 (8.5–12.5)
MELD score (Mean, SD)	27.5 ± 8.7
CTP score (Median, IQR)	13 (12–14)
28‐day mortality (%)	17 (42.5%)
90‐day mortality (%)	26 (65%)

Data are presented as mean ± SD, Median (IQR) or number (%).

CLIF‐OF, chronic liver failure organ failure; CLIF‐SOFA, chronic liver failure–sequential organ failure assessment; CTP, Child‐Turcotte‐Pugh; HBV, hepatitis B virus; HCV, hepatitis C virus; MELD score, model for end‐stage liver disease score; NASH, non‐alcoholic steatohepatitis; TLC, total leukocyte count.

**Table 2:** Comparison between patients of various grades of ACLF

Parameters	No ACLF (*n* = 10)	Grade 1 (*n* = 10)	Grade 2 (*n* = 10)	Grade 3 (*n* = 10)	All ACLF (Grades 1–3) (*n* = 30)	*P* value ACLF‐0 *vs* ACLF 1–3	*P* value for trend
Age, years	47.6 ± 8.7	47.6 ± 15.2	48.7 ± 5.9	41.4 ± 7.7	45.9 ± 10.6	0.65	0.36
Gender (M:F)	9:1	3:2	10:0	10:0	13:2		
Hemoglobin, gm/dL	9.0 ± 1.1	9.5 ± 2.4	8.8 ± 1.4	9.9 ± 2.6	9.4 ± 2.2	0.55	0.63
Platelet × 10^3^/mm^3^	140 ± 90	108 ± 75	118 ± 79	139 ± 80	120 ± 70	0.52	0.86
TLC × 10^3^/mm^3^	9.0 ± 3.9	13.3 ± 7.2	12.8 ± 8.5	18.4 ± 18.4	14.9 ± 12.2	**0.02**	0.30
Polymorphs	70.6 ± 6.6%	72.4 ± 13	80.3 ± 6.3	75.9 ± 12.0	76.2 ± 10.9%	0.14	0.15
INR	1.6 ± 0.3	2.1 ± 1.0	2.2 ± 1.5	3.2 ± 1.7	2.5 ± 1.3	**0.02**	**0.014**
Ascites	90%	80%	100%	100%	94%		
Creatinine, mg/dL	1.1 ± 0.4	1.5 ± 0.9	1.8 ± 1.1	2 ± 1.9	2.2 ± 2.1	0.10	**0.03**
Bilirubin, mg/dL	4.9 ± 2.9	18.2 ± 14.3	14.6 ± 9	27 ± 8.3	20.2 ± 11.9	**0.001**	**<0.001**
Total protein, gm/dL	6.3 ± 1	6.2 ± 1	5.7 ± 1	5.2 ± 1	5.7 ± 0.9	0.13	0.06
Albumin, gm/dL	2.4 ± 0.3	2.3 ± 0.5	2 ± 0.2	2.2 ± 0.3	2.2 ± 0.5	0.09	0.24
Duration of hospital stay (days)	4.5 ± 3.2	12.3 ± 5.7	11.6 ± 6	10 ± 7.9	11.3 ± 6.4	**0.003**	**0.026**
28‐day mortality	0%	40%	50%	80%	56.6%	**0.002**	**0.004**
90‐day mortality	20%	80%	70%	90%	80%	**0.001**	**0.005**

Data are presented as mean ± SD or as number (percentage).

ALT, alanine aminotransferase; AST, aspartate aminotransferase; CLIF‐OF, chronic liver failure‐organ failure score; CLIF‐SOFA, chronic liver failure–sequential organ failure assessment; INR, international normalized ratio; MELD score, model for end‐stage liver disease score; TLC, total leucocyte count.

#### *Neutrophil immunophenotype*

**Figure 2:**
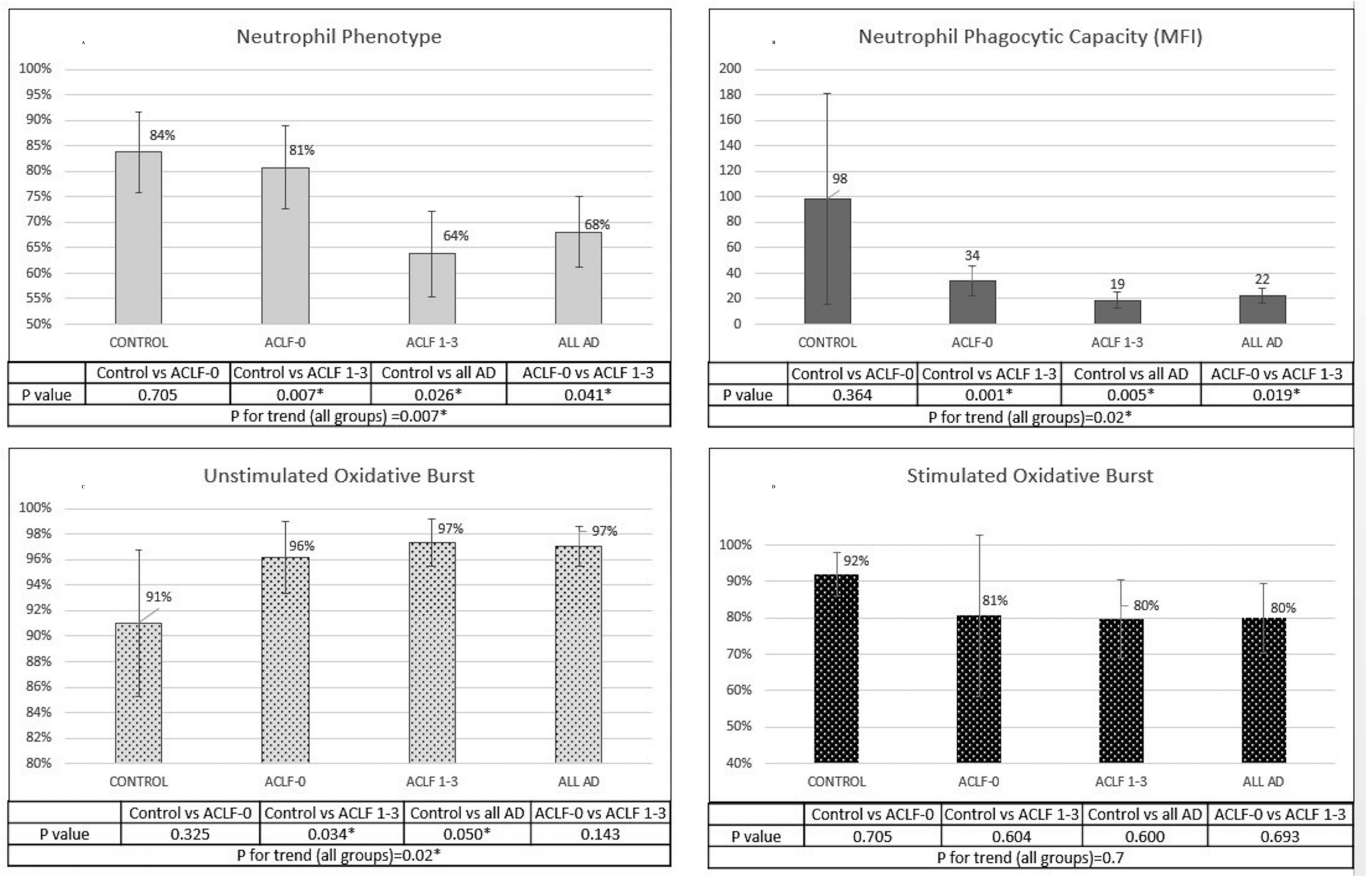
Neutrophil dysfunction among patients with acute decompensation (AD) of cirrhosis and acute on chronic liver failure (ACLF). (a) Neutrophil phenotype (NP): The proportion of phenotypically normal neutrophils (in %) at baseline was significantly reduced in all patients with AD compared to controls (*P* = 0.026). ACLF 1–3 had significantly lower phenotypically normal neutrophils compared to ACLF‐0 (*P* = 0.041) and controls (*P* = 0.007). No significant difference was seen between ACLF‐0 and controls (*P* = 0.700). (b) Neutrophil phagocytic capacity (NPC): Neutrophils from all AD patients had a trend toward impaired phagocytosis (in MFI) compared to controls (*P* = 0.098). ACLF 1–3 had significantly lower NPC compared to ACLF‐0 (*P* = 0.019) and controls (*P* = 0.001). No significant difference seen between ACLF‐0 and controls (*P* = 0.360). (c,d) Neutrophil oxidative burst (OB): Neutrophils from all AD patients had higher resting OB compared to controls (*P* = 0.05). In ACLF 1–3 groups, resting OB was higher compared to controls (*P* = 0.03). No significant difference seen between resting OB of patients with ACLF‐0 compared to ACLF 1–3 or controls. Stimulation with PMA did not lead to improved OB in patients with ACLF or AD of cirrhosis. No significant difference was seen among patients with ACLF‐0 compared to ACLF 1–3 or controls.

The proportion of phenotypically normal neutrophils (in %) at baseline was significantly reduced in all patients with AD compared to controls (68.05 ± 22.03 *vs* 83.74 ± 12.38; *P* = 0.026) (Fig. 2a). ACLF 1–3 had significantly lower phenotypically normal neutrophils compared to ACLF‐0 (63.84 ± 22.98 *vs* 80.70 ± 12.85; *P* = 0.041) and controls (*P* = 0.007). However, there was no significant difference between ACLF‐0 and controls (*P* = 0.700).

#### *Neutrophil phagocytic capacity*

Neutrophils isolated from patients of AD of cirrhosis at the time of enrolment had impaired phagocytosis (in MFI) compared to controls, although this was not statistically significant (98.33 ± 130.60 *vs* 22.47 ± 18.95; *P* = 0.098). Patients with ACLF 1–3 had significantly lower NPC compared to ACLF‐0 (18.73 ± 17.88 *vs* 33.69 ± 18.42; *P* = 0.019) and controls (18.73 ± 17.88 *vs* 98.33 ± 130.60; *P* = 0.001) (Fig. 2b). There was no significant difference seen among patients with ACLF‐0 and controls (*P* = 0.36).

#### *Neutrophil oxidative burst*

Patients with AD of cirrhosis had higher resting OB compared to controls (*P* = 0.05). In the ACLF groups, resting OB was higher compared to controls (*P* = 0.03) (Fig. 2c). There was no significant difference between resting OB of patients with ACLF‐0 compared to ACLF 1–3 or controls. Stimulation with PMA did not lead to increased OB in patients with ACLF or AD of cirrhosis (Fig. 2d). There was no significant difference seen among patients with ACLF‐0 compared to ACLF 1–3 or controls.

#### *Association of neutrophil dysfunction with patient outcomes*

**Figure 3:**
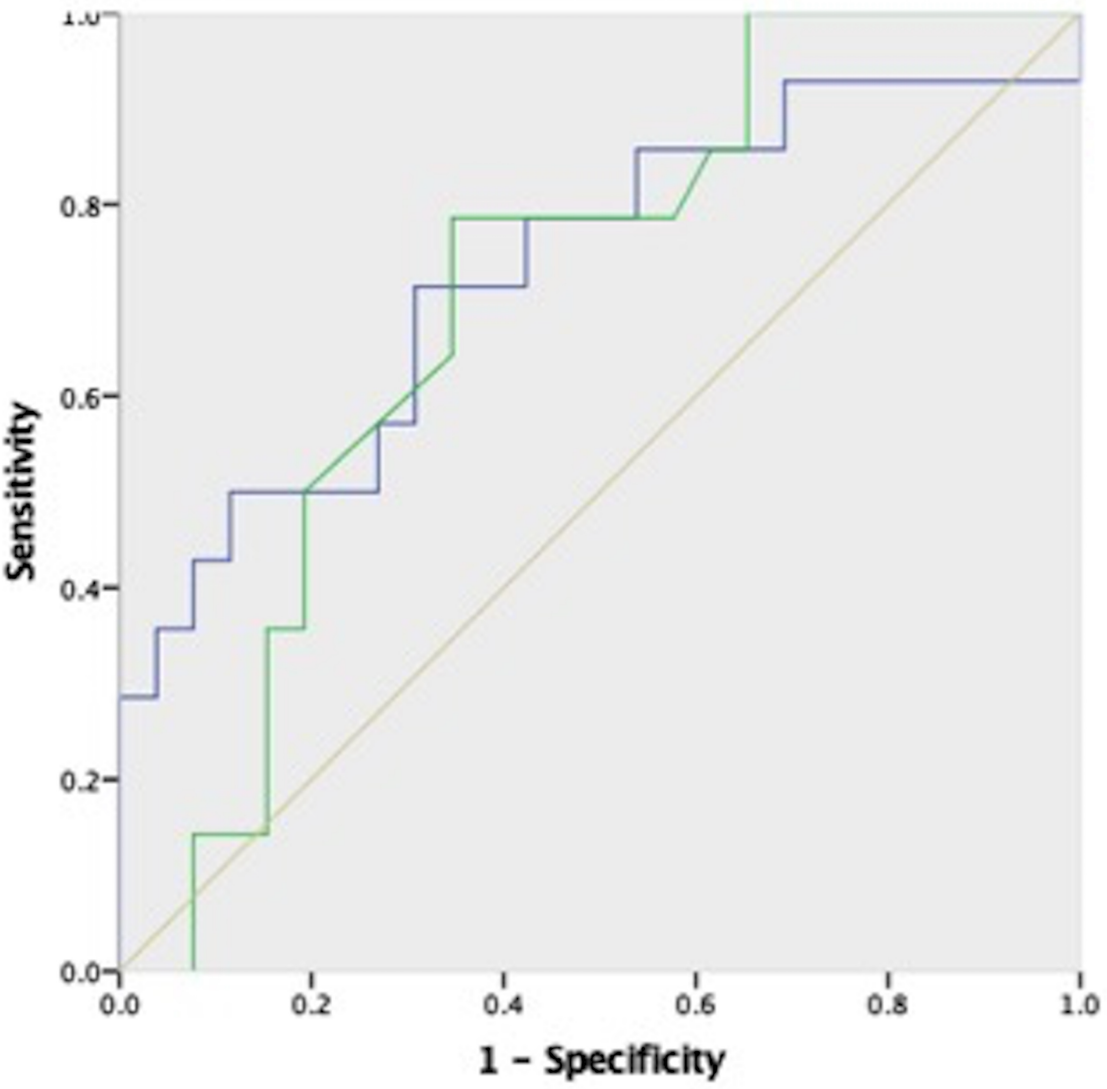
Area under receiver operating curve depicting neutrophil immunophenotype (NP; defined as % cells positive for both CD16 and 66b) and neutrophil phagocytic capacity (NPC) as predictors of 90‐day transplant‐free survival in acute decompensation of cirrhosis. Phenotypically normal neutrophils >71.7% had 78.6% sensitivity and 65.4% specificity with area under receiver operating curve (AUROC) of 0.70 (95% CI: 0.55–0.90), *P* = 0.017, and NPC >17.32. MFI had 71.4% sensitivity and 69.6% specificity with AUROC of 0.73 (95% CI: 0.54–0.86), *P* = 0.035, in predicting 90‐day survival. 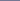, NPC; 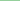, NP; 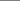, Reference line.

Overall 28‐day and 90‐day mortality was 42.5% and 65%, respectively, in all patients with AD, and none of the patients enrolled in the study underwent liver transplant. Among patients with ACLF, ACLF‐3 had maximum 90‐day mortality (90%). NP was normal (in %) and NPC (in MFI) was better in 90‐day survivors compared to nonsurvivors (78 ± 11.9 *vs* 62.2 ± 24.11, *P* = 0.02, and 33.3 ± 22.7 *vs* 16.36 ± 13.3; *P* = 0.004, respectively). Percentage of mature neutrophil (positive for both CD16 and CD66b) predicted 90‐day transplant‐free survival in patients with AD of cirrhosis (AUROC = 0.705; *P* = 0.035). Percentage of mature neutrophil (positive for both CD16 and CD66b) of more than 71.7% predicted 90‐day survival with 78.6% sensitivity and 65.4% specificity. NPC also predicted 90‐day survival in patients with AD of cirrhosis (AUROC = 0.731; *P* = 0.017). NPC of more than 17.32 MFI predicted 90‐day survival with 71.4% sensitivity and 69.6% specificity. No difference was observed in OB between 90‐day survivors and nonsurvivors (Fig. 3).

During hospitalization, infection was clinically suspected in 11 (26.8%) patients. The incidence of culture‐positive sepsis was low among patients of ACLF. While one patient of grade 2 ACLF developed *Pseudomonas aeruginosa*‐related spontaneous bacterial peritonitis on day 4, another patient of grade 3 ACLF developed *Escherichia coli*‐related blood stream infection. Patients with infection were found to have higher 28‐day (*P* = 0.002) and 90‐day mortality (*P* = 0.004).

### *Etiological considerations*

We compared the degree of neutrophil dysfunction between patients with and without acute alcoholic hepatitis. There was no significant difference in NP (in %) (64.9 ± 24.5 *vs* 70.4 ± 20.1, *P* = 0.44), resting OB (in %) (709.9 ± 851.2 *vs* 901.1 ± 925.3, *P* = 0.50), stimulated OB (in %) (5487.9 ± 3741.4 *vs* 5283.2 ± 3874.8, *P* = 0.86), or NPC (23.1 ± 17.7 *vs* 22.0 ± 20.2, *P* = 0.85).

## Discussion

This study suggest a severe functional failure of neutrophils in patients with ACLF despite increase in number of polymorphs in patients with ACLF, which was associated with increased 90‐day mortality. Neutrophil dysfunction was characterized with impaired expression of surface receptors and phagocytic potential, suggesting a reduced capacity for mounting an effective innate immune response against exogenous pathogens. Ironically, this was seen in addition to an increased background OB of the neutrophils, which led to a generalized proinflammatory state.

Human neutrophils are an important component of innate immunity and forms a first‐line defense against microbial infections. However, paradoxically, they also play a role in host cell injury in many diseased states, such as alcoholic hepatitis.^6^ Neutrophil surface expression of cluster of differentiation (CD) antigens may be used to identify functionally normal mature neutrophils. Lakschevitz *et al*.^12^ showed that a combination of CD markers (CDD11b, CD16, and CD66b) was able identify mature and phenotypically normal cells that were used in our study. CD 11b, CD16, and CD66b are involved in cellular adhesion, transendoethelial migration, and phagocytosis, respectively. In our study, neutrophils showed reduced expression of CD16 and CD66b, which may be associated with impaired phagocytosis in patients with ACLF compared to controls. Previous studies^9^ failed to show a significant decrease in NP in patients with ACLF. The presence of occult sepsis could have been a confounding factor in those studies as it can lead to increased neutrophil expression of CD66b and CD16 through emergency granulopoiesis, thereby leading to a higher number of phenotypically normal neutrophils.^15^ Ours is the only study to exclude infection at baseline, thereby isolating the neutrophil dysfunction of ACLF from sepsis.

There is a marked increase in oxygen consumption, termed “respiratory burst,” occurring during phagocytosis.^6^ We showed that neutrophils in patients with ACLF were in a state of sustained heightened OB at rest. However, after stimulation with PMA, OB failed to increase, signifying a defective response of neutrophils,^13^ which was further supported by their decreased phagocytic capacity. This functional defect can be explained by a state of persistent stimulation of neutrophils at rest in patients with ACLF by inflammatory factors or lipopolysaccharides(LPS), which are often the result of bacterial translocation of gut flora into portal circulation, which eventually leads to neutrophil exhaustion.^16^ OB variation with liver disease severity has shown different results in various studies.^9^, ^17^ We speculate that OB in vivo is influenced by a number of factors, such as ammonia, cytokines, and infection, and with ACLF being a dynamic syndrome, these factors go on to change and sometimes do not necessarily correlate with liver disease severity.^9^ Removal of endotoxins, cytokines, and ammonia via plasma exchange is an exciting opportunity to reverse the neutrophil dysfunction in patients with ACLF.^9^

The paradox of increased OB and systemic inflammation but reduced NPC in ACLF could be explained by the increased secretion and activation of neutrophil elastase (due to ROS‐mediated inhibition of protease inhibitor, which cleaves elastase).^18^ Elevated elastase enzyme cleaves opsonin receptor CR1 and chemokine receptor CXCR1 on the neutrophil surface, which in turn reduces the opsonin‐mediated phagocytosis.^18^ Moreover, increased elastase can induce tissue destruction and further inflammation in cirrhosis.^19^ Energy depletion due to high OB may also reduce host defense mechanisms, including NPC.^9^

We have also shown the clinical importance of this neutrophil dysfunction, which was identified by a phagocytic capacity of more than 17.3 MFI and NP >71.7% and was able to predict patient survival in patients with ACLF. This information can be of great clinical significance when coupled with evidence on neutrophil stimulation by granulocyte colony‐stimulating factors (G‐CSF). However, this dysfunction is reversible, and this reversibility has been shown to affect the outcome in these patients.^14^ Measures to bring about this reversibility can improve the outcome in these patients.

There is emerging evidence of the use of G‐SCF in patients with ACLF and decompensated cirrhosis leading to improved survival.^20^, ^21^ While opinion on the efficacy of this therapy is divided, a careful review of the studies show that the maximum difference in mortality comes in the initial few weeks of treatment. It has been postulated that this benefit is derived from the quantitative and qualitative effects of G‐CSF on neutrophils. In addition to the obvious increase in neutrophil numbers, G‐CSF seems to enhance the function of normal and dysfunctional neutrophils.^22^ Our study supports the school of thought that stimulating bone marrow^20^, ^21^ to release new neutrophils during the window period^2^ could potentially alter patient outcome. This study paves the way for further trials where growth factor support can be given selectively to patients with poor neutrophil function and, thus, poor prognosis.

We recognize that, despite attempts to exclude all patients with pre‐existing infections at the baseline, we cannot fully eliminate the possibility of underlying subclinical infections that were not detected by current routine microbiology techniques, which might have precipitated a high resting OB in some patients. The other limitation of our study was the small numbers in each group.

In conclusion, our study provides important clues to show that neutrophils in patients with ACLF are in a “burned out” or “exhausted” state. The association between neutrophil dysfunction and poor survival in different grades of ACLF requires further studies. Currently, these neutrophil function tests are cumbersome to perform, but as evidence evolves, simpler tests for the assessment of neutrophil dysfunction may strengthen the estimation of prognosis of patients with ACLF.

## Declaration of conflict of interest

The authors have nothing to disclose. The study was supported by Postgraduate Institute of Medical Education & Research, Chandigarh.

## Author contributions

Radha K Dhiman conceptualized the study design, interpreted the data, performed critical revisions of manuscript, and provided administrative support and supervision. Shallu Tomer conducted laboratory experiments and analysis of data and drafted the manuscript. Nipun Verma interpreted the data, drafted the manuscript, and performed critical revisions of manuscript. Sahaj Rathi performed critical revisions of manuscript. Sunil K Arora conducted analysis and supervised the laboratory experiments. Sunil Taneja, Ajay Duseja, and Yogesh K Chawla performed patient management, follow up, and study supervision.
